# Inhibition of deubiquitinases primes glioblastoma cells to apoptosis *in vitro* and *in vivo*

**DOI:** 10.18632/oncotarget.7302

**Published:** 2016-02-10

**Authors:** Georg Karpel-Massler, Matei A. Banu, Chang Shu, Marc-Eric Halatsch, Mike-Andrew Westhoff, Jeffrey N. Bruce, Peter Canoll, Markus D. Siegelin

**Affiliations:** ^1^ Department of Pathology and Cell Biology, Columbia University Medical Center, New York, New York, USA; ^2^ Department of Neurosurgery, Columbia University Medical Center, New York, New York, USA; ^3^ Department of Neurosurgery, Ulm University Medical Center, Ulm, Germany; ^4^ Department of Pediatrics and Adolescent Medicine, Ulm University Medical Center, Ulm, Germany

**Keywords:** apoptosis, deubiquitinases, Bcl-2 family proteins, glioblastoma

## Abstract

It remains a challenge in oncology to identify novel drug regimens to efficiently tackle glioblastoma, the most common primary brain tumor in adults. Here, we target deubiquitinases for glioblastoma therapy by utilizing the small-molecule inhibitor WP1130 which has been characterized as a deubiquitinase inhibitor that interferes with the function of Usp9X. Expression analysis data confirm that Usp9X expression is increased in glioblastoma compared to normal brain tissue indicating its potential as a therapeutic. Consistently, increasing concentrations of WP1130 decrease the cellular viability of established, patient-derived xenograft (PDX) and stem cell-like glioblastoma cells. Specific down-regulation of Usp9X reduces viability in glioblastoma cells mimicking the effects of WP1130. Mechanistically, WP1130 elicits apoptosis and increases activation of caspases. Moreover, WP1130 and siRNAs targeting Usp9X reduce the expression of anti-apoptotic Bcl-2 family members and Inhibitor of Apoptosis Proteins, XIAP and Survivin. Pharmacological and genetic interference with Usp9X efficiently sensitized glioblastoma cells to intrinsic and extrinsic apoptotic stimuli. In addition, single treatment with WP1130 elicited anti-glioma activity in an orthotopic proneural murine model of glioblastoma. Finally, the combination treatment of WP1130 and ABT263 inhibited tumor growth more efficiently than each reagent by its own *in vivo* without detectable side effects or organ toxicity. Taken together, these results suggest that targeting deubiquitinases for glioma therapy is feasible and effective.

## INTRODUCTION

Glioblastoma is among the most difficult to treat human malignancies and consequently patients diagnosed with a glioblastoma succumb to this disease within 2 years in the vast majority of cases [1]. The lack of therapeutic progress stands in sharp contrast to a fast-paced increase in knowledge about the molecular nature of this disease. Among the many factors that are responsible for the recalcitrant behavior of these neoplasms is the heterogeneity of gliomas which remains to be one of the most challenging features to target. Within a single glioblastoma tumor many pathways are dysregulated even at the single-cell level which underlines the necessity for the combination of different targeted agents in a multi-targeting approach [2].

Malignant transformation and maintenance of the neoplastic cellular phenotype are the result of dysregulation of a complex molecular interplay. Within this molecular framework, post-translational modification such as ubiquitination/deubiquitination represents a major determinant for protein stability, trafficking or function and is therefore conceived to be key to confer the balance between pro- and anti-apoptotic signaling [3]. Once this complex system is dysregulated cells are potentially guided towards a neoplastic phenotype and the formation of cancer. As a consequence, small molecule inhibitors such as the deubiquitinase inhibitor WP1130 were designed to allow for a restoration of cellular homeostasis as potential anti-cancer agents.

The deubiquitinating enzyme Usp9X was shown to be overexpressed in various human cancers, including glioblastoma (Supplementary Figure S1), which supports the notion that deubiquitinating enzymes represent a valid target in this disease and that their inhibition may add to a concerted strategy against glioblastoma [4]. Consequently, we show in this study that interference with deubiquitinases, such as Usp9X, results in a marked anti-proliferative and pro-apoptotic effect on established, glioma stem-like and primary patient-derived xenograft glioblastoma cells. Morever, this effect can be synergistically enhanced when combined with the BH3-mimetic ABT263 or the death receptor ligand Tumor necrosis factor α-related apoptosis-inducing ligand (TRAIL). More importantly, we show that local intracranial administration of the deubiquitinase inhibitor WP1130 using convection-enhanced delivery results in a prolonged survival *in vivo*. In addition, when combining WP1130 with ABT263 tumor growth is significantly attenuated compared to single-agent treatment or control *in vivo*.

## RESULTS

### The deubiquitinase Usp9X is highly expressed in glioblastoma

*In silico* analysis revealed that DNA copy number or mRNA expression of Usp9X is significantly increased in glioblastoma and anaplastic astrocytoma when compared to normal brain (Supplementary Figure S1). Moreover, when analyzing the Rembrandt database, patients carrying less than 1.8 copies of the Usp9X gene seemed to have a better prognosis with respect to overall survival (Supplementary Figure S2).

### Treatment with the deubiquitinase inhibitor WP1130 inhibits proliferation of established glioblastoma and glioma stem-like cells

**Figure 1:**
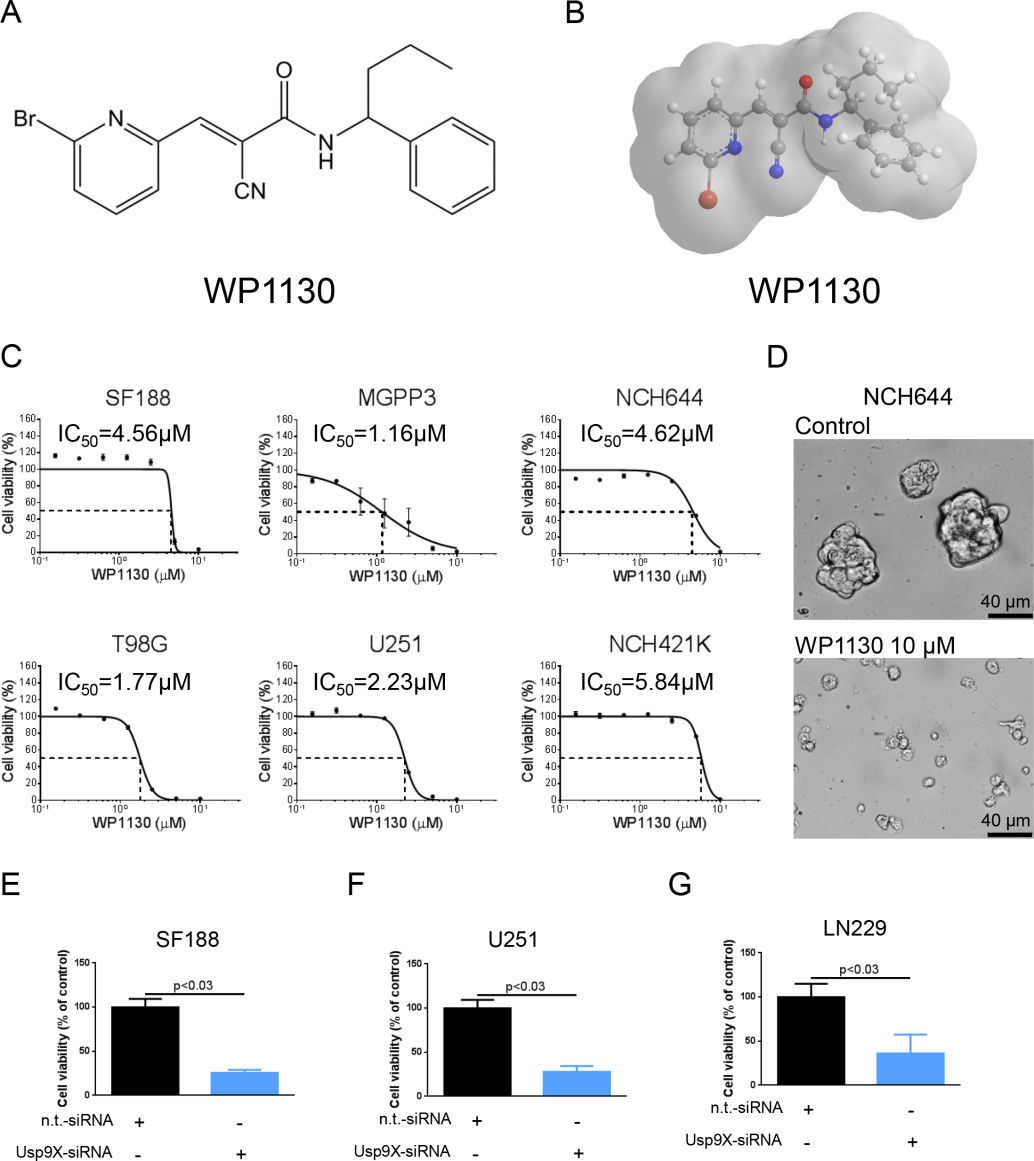
Interference with deubiquitinase activity inhibits proliferation across different glioblastoma cells (**A**) Chemical structure of the deubiquitinase inhibitor WP1130. (**B**) 3-dimensional representation of the deubiquitinase inhibitor WP1130. (**C**) SF188 (pediatric), T98G (adult), MGPP-3 (murine, transgenically-derived), U251 (adult) glioblastoma cells and NCH644, NCH421K glioma stem-like cells were treated with increasing concentrations of WP1130 under serum starvation (1.5% FBS). After 72 h, MTT assays were performed. Dose-response curves and IC_50_-values were calculated using non-linear regression. Data are presented as mean and SEM (**D**) Representative microphotographs of NCH644 glioma stem-like cells treated with solvent or WP1130 for 48 h at indicated concentrations. Magnification, ×40; scale bar, 40 μm. (**E**–**G**) SF188 (E), U251 (F) and LN229 (G) glioblastoma cells were transfected with Usp9X-siRNA. MTT assays were performed after 72 h to detect anti-proliferative effects. Columns, means. Bars, SD.

To assess whether inhibition of deubiquitinases affects proliferation of glioblastoma cells *in vitro* we treated SF188 (pediatric), MGPP-3 (proneural, transgenic), T98G and U251 glioblastoma cells as well as NCH644 and NCH421K glioma stem-like cells with increasing concentrations of the deubiquitinase inhibitor WP1130 (Figure 1A and 1B) prior to performing MTT assays. As shown in Figure 1C, treatment with WP1130 yielded an anti-proliferative effect across all cell lines tested in a dose-dependent manner. Notably, treatment with WP1130 resulted in marked anti-proliferative activity and morphological changes in NCH644 and NCH421K glioma stem-like cells (Figure 1C and 1D, Supplementary Figure S3A).

### Down-regulation of Usp9X inhibits proliferation in glioblastoma cell lines

In order to further examine whether the anti-proliferative effect on glioblastoma cells following a treatment with WP1130 can be recapitulated by specific knock-down of Usp9X we performed siRNA experiments. As shown in Figure 1E–1G, treatment with Usp9X-siRNA resulted in significantly reduced cell viability. Specific knock-down was confirmed via Western blot analysis (Figures 3B and 4F).

### Treatment with the deubiquitinase inhibitor WP1130 induces apoptosis in glioblastoma cells

**Figure 2:**
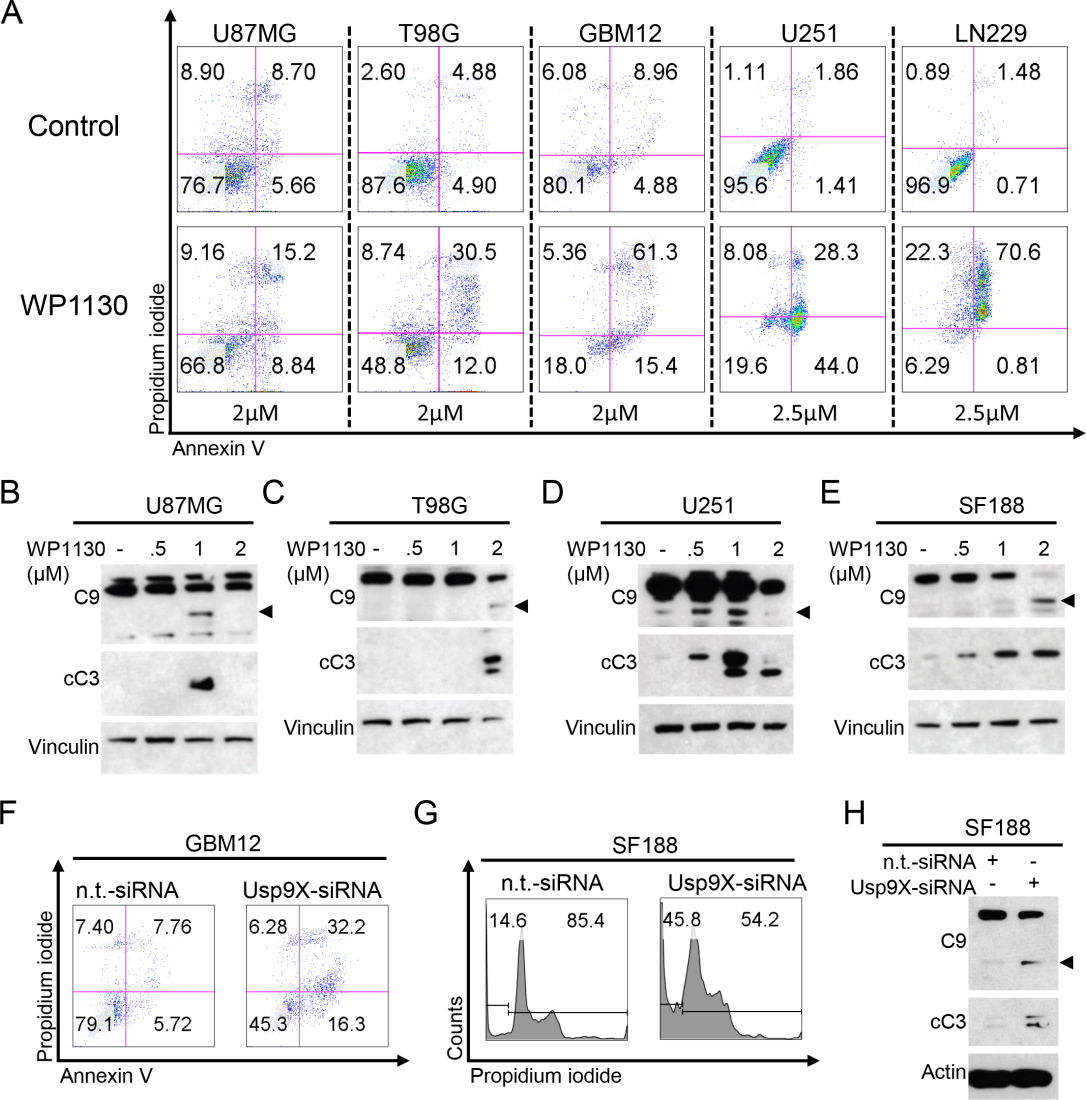
Inhibition of deubiquitinases induces apoptosis in glioblastoma (**A**) representative flow plots of U87MG, T98G, GBM12, U251 and LN229 glioblastoma cells that were treated as indicated with WP1130 prior to staining with annexin V/propidium iodide (PI) and flow cytometric analysis. (**B**–**E**) U87MG (B), T98G (C), U251 (D) and SF188 (E) glioblastoma cells were treated for 6 h with increasing concentrations of WP1130 under serum starvation (1.5% FBS). Whole-cell extracts were examined by Western blot for caspase 9 (C9 – arrow head = cleaved fragment) and cleaved caspase 3 (cC3). Vinculin Western blot analysis was performed to confirm equal protein loading. (**F**) Representative flow plots of GBM12 glioblastoma cells transfected either with n.t.-siRNA or Usp9X-siRNA and stained for annexin V/PI prior to flow cytometric analysis. (**G**) Representative flow plots of SF188 glioblastoma cells transfected either with n.t.-siRNA or Usp9X-siRNA and stained for PI prior to flow cytometric analysis. (**H**) SF188 glioblastoma cells were transfected with n.t.-siRNA or Usp9X-siRNA. Whole-cell extracts were examined by Western blot for caspase 9 (C9 – arrow head=cleaved fragment) and cleaved caspase 3 (cC3). Actin Western blot analysis was performed to confirm equal protein loading.

Next, we assessed if apoptosis may be responsible for the antiproliferative effect of deubiquitinase inhibition we observed. Therefore, we treated established glioblastoma cell lines and patient-derived GBM12 glioblastoma cells with WP1130 and determined the fluorescent intensity of annexin V and propidium iodide (PI). In all glioblastoma cells we tested the fraction of annexin V-positive and PI-negative cells (early apoptotic cells) and/or the fraction of annexin V- and PI-positive cells (late apoptotic cells) was significantly increased when treated with WP1130 (Figure 2A, Supplementary Figure S3B). In concordance with these data, Western blot analyses showed that cleavage of caspases 9 and 3 was also markedly enhanced following treatment of U87MG (B), T98G (C), U251 (D) and SF188 (E) glioblastoma cells with increasing concentrations of WP1130 (Figure 2B–2E).

### Specific knock-down of Usp9X induces apoptosis in glioblastoma cells

We next examined whether specific knock-down of Usp9X recapitulates the pro-apoptotic effect caused by treatment with WP1130. Therefore, we performed siRNA experiments down-regulating protein expression of Usp9X in GBM12 patient-derived glioblastoma cells and SF188 pediatric glioblastoma cells. As shown in Figure 2F and 2G, the fraction of apoptotic cells was significantly increased in both cell lines. On the molecular level, cleavage of caspases 9 and 3 was significantly increased when Usp9X was down-regulated (Figure 2H).

### Inhibition of deubiquitinases decreases the expression of anti-apoptotic Bcl-2 family members and the Mcl-1 chaperone, Bag3

**Figure 3:**
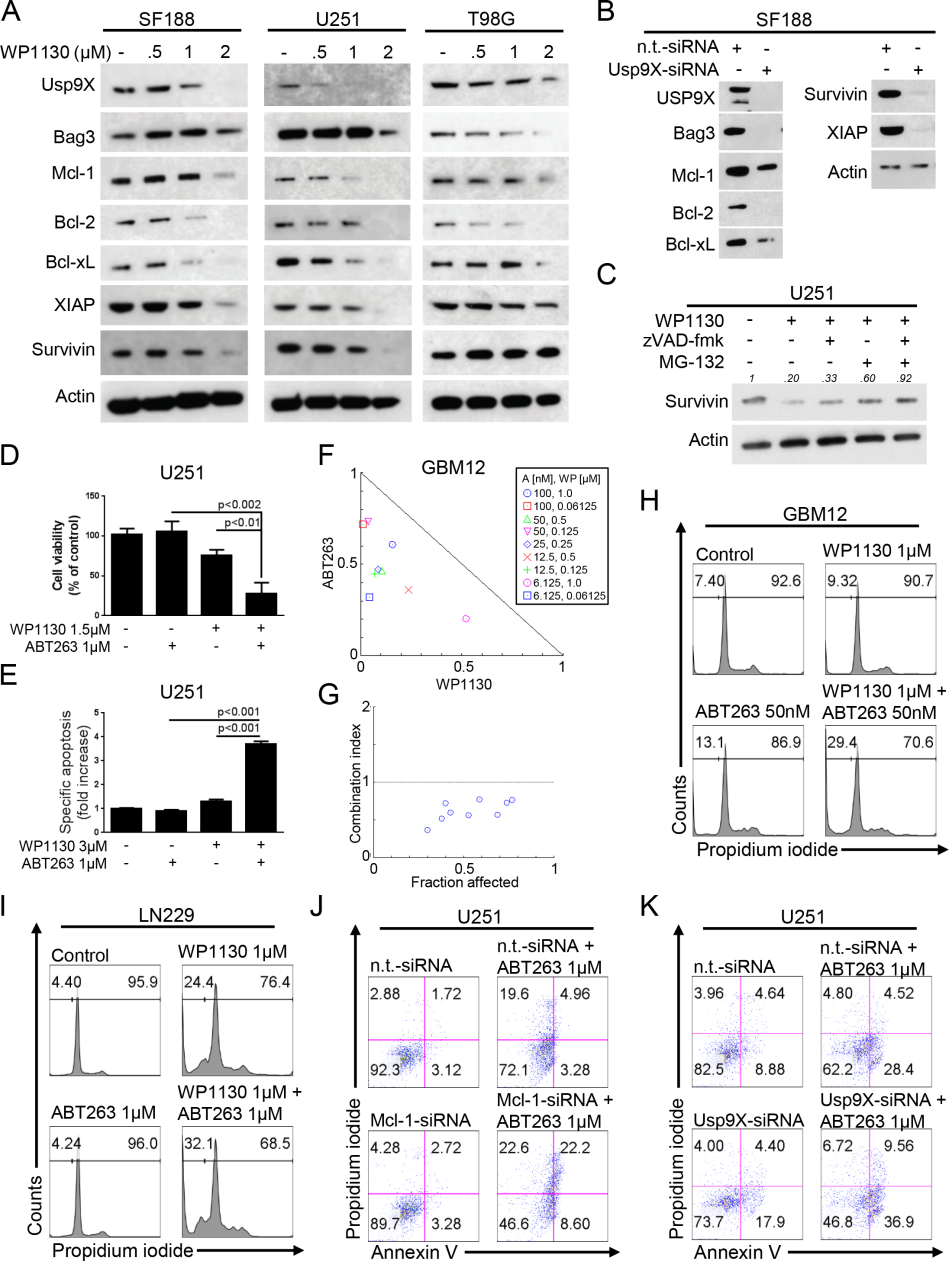
Inhibition of deubiquitinases yields down-regulation of the Mcl-1/Bag3/Usp9X-axis and sensitizes for the BH3-mimetic ABT263 (**A**) SF188, U251 and T98G glioblastoma cells were treated for 24 h with increasing concentrations of WP1130 under serum starvation. Whole-cell extracts were examined by Western blot for Usp9X, Bag3, Mcl-1, Bcl-2, Bcl-xL, XIAP and Survivin. Actin served as a loading control. (**B**) SF188 glioblastoma cells were treated either with n.t.-siRNA or Usp9X-siRNA. Whole-cell extracts were examined by Western blot for Usp9X, Bag3, Mcl-1, Bcl-2, Bcl-xL, Survivin and XIAP. Actin Western blot analysis was performed to confirm equal protein loading. (**C**) U251 glioblastoma cells were treated for 5 h with WP1130 (2.5 μM), zVAD-fmk (20 μM) and MG-132 (10 μM) as indicated. Whole cell extracts were collected and Western blot analysis for Survivin was performed. Actin served as a loading control. Densitometric analysis was performed using ImageJ (National Institutes of Health, U.S.A., http://imagej.nih.gov/ij). Data were normalized first to the respective actin control and second to the respective treatment control. (**D**) U251 glioblastoma cells were treated for 72 h with ABT263, WP1130 or both. Cellular viability was determined by performing MTT assays. (**E**) U251 glioblastoma cells were treated for 48h with ABT263, WP1130 or both as indicated. Staining for annexin V/propidium iodide (PI) was performed prior to flow cytometric analysis. The fraction of annexin V- and annexin V/PI-positive cells was determined and expressed as fold increase versus control. Columns, means. Bars, SD. (**F**) The antiproliferative effect of ABT263 (A) and WP1130 (WP) was assessed by an MTT assay after 72 h of treatment with the single agents or the respective combination at indicated concentrations under serum starvation (1.5% FBS) in GBM12 glioblastoma cells. Normalized isobolograms were calculated using the CompuSyn software. The connecting line represents additivity. Data points located below the line indicate a synergistic drug-drug interaction and data points above the line indicate an antagonistic drug-drug interaction. (**G**) Graphical representation of the combination index for the data points shown under F. The horizontal line, signifying a combination index equal of 1, represents additivity. Data points below indicate synergism; data points above indicate antagonism. (**H**–**I**) Representative flow plots of GBM12 (H) and LN229 (I) glioblastoma cells that were treated with WP1130 and/or ABT263 as indicated prior to staining with PI and flow cytometric analysis. (**J**) U251 glioblastoma cells were treated with non-targeting (n.t.)-siRNA, or Mcl-1-siRNA followed by a treatment with ABT263 or solvent for 24 h. Staining for annexin V/PI was performed prior to flow cytometric analysis. Representative flow plots are shown. (**K**) U251 glioblastoma cells were treated with non-targeting (n.t.)-siRNA, or Usp9X-siRNA followed by a treatment with ABT263 or solvent for 24 h. Staining for annexin V/PI was performed prior to flow cytometric analysis. Representative flow plots are shown.

Anti-apoptotic Bcl-2 family proteins are important regulators of apoptosis and known to confer resistance to anti-cancer treatments. The deubiquitinase Usp9X and the chaperone Bag3 stabilize the anti-apoptotic Bcl-2 family member Mcl-1. Since WP1130 inhibits Usp9X we further assessed the effects of WP1130 treatment on Mcl-1 as well as Bcl-2 and Bcl-xL protein expression. As anticipated, treatment with WP1130 lead to down-regulation of Usp9X in a dose-dependent manner in SF188, U251 and T98G glioblastoma cells (Figure 3A). In addition, expression of Bag3 was also markedly reduced. Consistent with these findings, Mcl-1 expression was decreased in all studied cell lines. Moreover, Bcl-2 and Bcl-xL expression was also significantly decreased upon treatment with WP1130 in a dose-dependent manner. These molecular alterations following a treatment with WP1130 were mirrored when silencing Usp9X with siRNA in SF188 pediatric glioblastoma cells (Figure 3B).

### Down-regulation of Mcl-1 and Usp9X occurs through a post-transcriptional mechanism

To assess whether the WP1130-mediated down-regulation of Mcl-1 and Usp9X is due to a transcriptional mechanism we performed real-time PCR analyses in U251 glioblastoma cells following treatment with WP1130. As shown in Supplementary Figure S4, treatment with WP1130 resulted in a dose-dependent and probably compensatory increase of Mcl-1 mRNA levels after 6 h and only a minor decrease after 24 h. Similar findings were observed for Usp9X levels. These data suggest that WP1130 down-regulates Mcl-1 and Usp9X through a post-transcriptional mechanism.

### Interference with deubiquitinases suppresses the levels of the anti-apoptotic inhibitor of apoptosis proteins, survivin and XIAP

Because IAPs are important regulators of apoptosis at the level of effector caspases, we determined the levels of the two most prominent members out of this family, Survivin and XIAP, in response to increasing concentrations of WP1130. We found that WP1130 suppressed the levels of XIAP and Survivin in SF188 and U251 cells further enhancing the effects of WP1130 on apoptosis (Figure 3A). These findings were recapitulated in SF188 cells silenced for Usp9X (Figure 3B). To assess whether WP1130-mediated down-regulation of Survivin is due to caspase-cleavage, U251 glioblastoma cells were treated with WP1130 in the presence or absence of the pan-caspase inhibitor zVAD-fmk (Figure 3C). Caspase inhibition resulted as anticipated in a partial, relatively minor rescue of Survivin protein expression. Since zVAD-fmk only partially restored Survivin levels, we examined to which extent inhibition of proteasomal degradation would counteract the loss in Survivin. Treatment with the proteasome inhibitor MG-132 yielded a stronger restoration of Survivin levels when compared to inhibition of caspases alone. This finding was even more pronounced when cells were treated with WP1130 in the presence of both zVAD-fmk and MG-132. Overall, these findings suggest that the decrease in Survivin is at least in part mediated by caspase-cleavage, but still mainly through enhanced proteasomal degradation.

### Inhibition of deubiquitinases sensitizes for the BH3-mimetic ABT263

**Figure 4:**
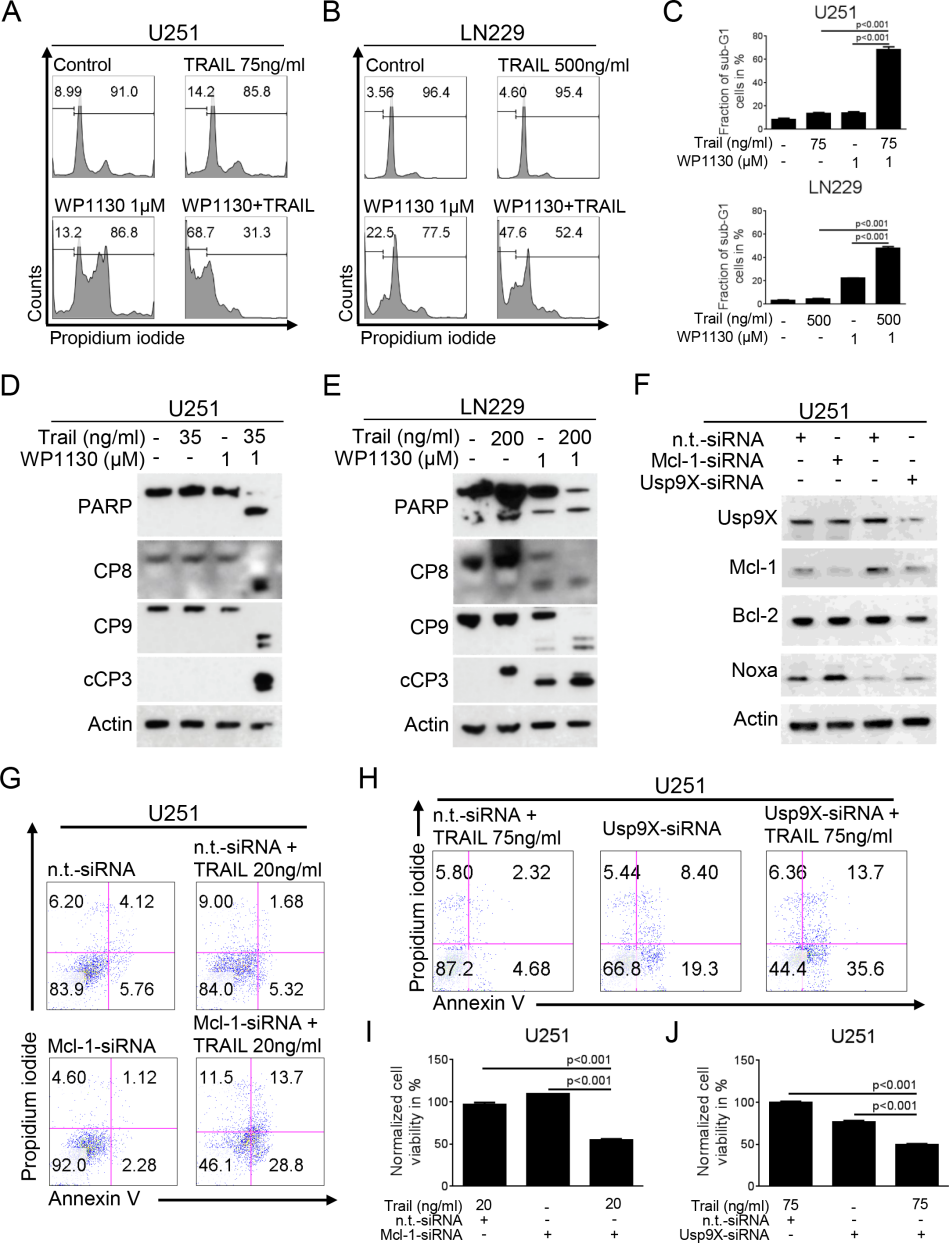
Inhibition of deubiquitinases sensitizes for TRAIL-mediated apoptosis A-B, U251 (**A**) and LN229 (**B**) glioblastoma cells were treated with WP1130 and/or TRAIL as indicated. Staining for propidium iodide (PI) was performed prior to flow cytometric analysis. Representative flow plots are shown. (**C**) Quantitative representation of cells treated as described for A-B (% sub-G1 cells). Columns, means. Bars, SD. D-E, U251 (**D**) and LN229 (**E**) glioblastoma cells were treated with WP1130 and/or TRAIL as indicated. Western blot analysis was performed for PARP, caspase 8 (CP8), caspase 9 (CP9) and cleaved caspase 3 (cCP3). Actin expression was determined to confirm equal protein loading. (**F**) U251 glioblastoma cells were transfected with n.t.-siRNA, Mcl-1-siRNA or Usp9X-siRNA. Whole-cell extracts were collected prior to Western blot analysis for Usp9X, Mcl-1, Bcl-2 and Noxa. Actin served as a loading control. (**G**) U251 glioblastoma cells were treated with non-targeting (n.t.)-siRNA, or Mcl-1-siRNA followed by a treatment with TRAIL or solvent for 24 h. Staining for annexin V/PI was performed prior to flow cytometric analysis. Representative flow plots are shown. (**H**) U251 glioblastoma cells were treated with non-targeting (n.t.)-siRNA, or Usp9X-siRNA followed by a treatment with TRAIL or solvent for 24 h. Staining for annexin V/PI was performed prior to flow cytometric analysis. Representative flow plots are shown. (**I**) Quantitative representation of the fraction of annexin V-positive and annexin V/PI-positive U251 cells treated as described for G. Columns, means. Bars, SD. (**J**) Quantitative representation of the fraction of annexin V-positive and annexin V/PI-positive U251 cells treated as described for H. Columns, means. Bars, SD.

Expression of Mcl-1 represents one of the most important mechanisms of resistance towards the BH3-mimetic ABT263. Since treatment with WP1130 results in a marked down-regulation of Mcl-1 we hypothesized that this finding should lead to an enhanced anti-neoplastic activity of ABT263 in a combinatorial treatment approach. We therefore treated U251, GBM12 and LN229 glioblastoma cells with WP1130, ABT263 or the combination prior to assessing anti-proliferative or pro-apoptotic activity. In all cell lines tested, we found a synergistic anti-proliferative and pro-apoptotic effect of the combination treatment (Figure 3D–3I) and (Table 1). Moreover, this observation was recapitulated by specific knock-down of either Mcl-1 (Figure 3J and Figure 4F) or Usp9X (Figure 3K and Figure 4F) when comparing knock-down plus ABT263 treatment to knock-down or ABT263 treatment alone.

**Table 1:** Combined treatment with ABT263 and WP1130 results in a synergistic anti-proliferative effect in GBM12 glioblastoma cells

ABT263 [nM]	WP1130 [μM]	CI
100.0	1.0	0.76505
100.0	0.06125	0.72979
50.0	0.5	0.57196
50.0	0.125	0.77058
25.0	0.25	0.56188
12.5	0.5	0.59853
12.5	0.125	0.51732
6.125	1.0	0.72265
6.125	0.06125	0.36340

The CompuSyn software (ComboSyn, Inc., Paramus, NJ, U.S.A.) was used for the drug-drug interaction analysis including the calculation of the combination index (CI). A CI < 1 was considered as synergistic, a CI = 1 as additive and a CI > 1 as antagonistic.

### Interference with deubiquitinases sensitizes for extrinsic apoptosis

To further address the question whether inhibition of deubiquitinases may also enhance inducers of extrinsic apoptosis we treated glioblastoma cells with TRAIL, WP1130 or the combination of both. As shown in Figure 4A–4C, combined treatment with TRAIL and WP1130 resulted in a synergistic pro-apoptotic effect on glioblastoma cells. In concordance with these findings, on the molecular level, the combination treatment resulted in enhanced cleavage of caspases 8, 9 and 3 as well as PARP in U251 and LN229 glioblastoma cells (Figure 4D and 4E).

### Knock-down of Mcl-1 or Usp9X sensitizes for TRAIL-mediated apoptosis

We showed that interference with deubiquitinases by treatment with WP1130 down-regulates Usp9X and Mcl-1. To assess whether down-regulation of Usp9X or Mcl-1 is sufficient to recapitulate the sensitizing effect of WP1130 for TRAIL-mediated apoptosis U251 glioblastoma cells were transfected with Mcl-1- or Usp9X-siRNA (Figure 4F). Both, knock-down for Mcl-1 (Figure 4G and 4I) and Usp9X (Figure 4H and 4J) resulted in a significant increase in the fraction of apoptotic cells when combined with TRAIL.

### Local delivery of WP1130 extends survival *in vivo*

**Figure 5:**
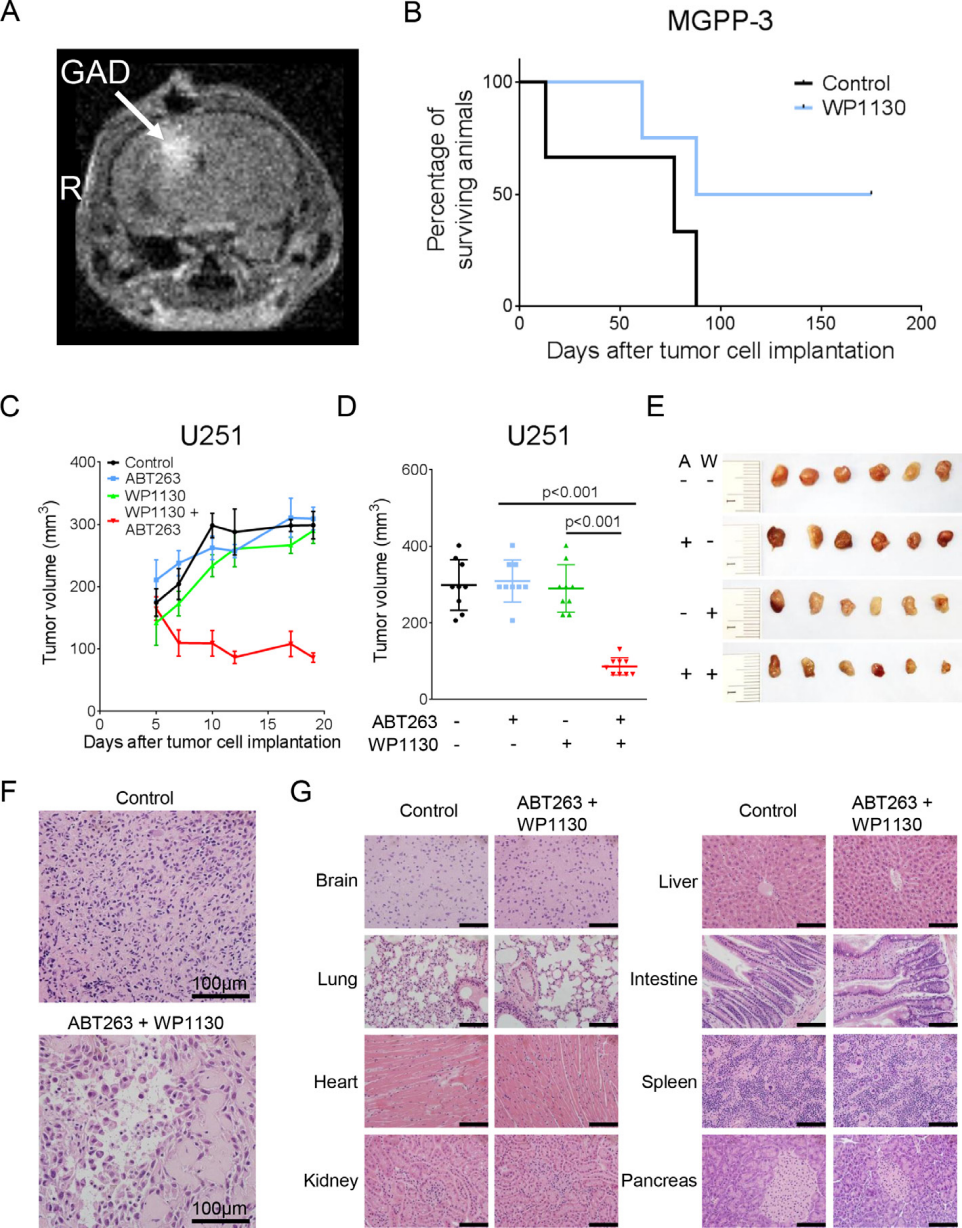
Intracranial convection-enhanced delivery of WP1130 prolongs survival and combined treatment with ABT263 and WP1130 results in an enhanced inhibition of tumor growth *in vivo* (A–B) 25000 MGPP-3 glioblastoma cells were implanted intracranially. After two weeks micro-osmotic pumps were implanted to either convect WP1130 (*n* = 4) or vehicle (*n* = 3) over 7 consecutive days. MRI imaging was performed to detect Gadolinium signal to verify the correct drug delivery. (**A**) Representative MRI image showing contrast enhancement; GAD = Gadolinium, R = right side. (**B**) Kaplan-Meier curves showing survival of animals treated either with vehicle or WP1130. C-G, 1 × 10^6^ U251 glioblastoma cells were implanted subcutaneously. After tumor formation animals were treated intraperitoneally with vehicle (*n* = 9 tumors), ABT263 (25 mg/kg; *n* = 9 tumors), WP1130 (25 mg/kg; *n* = 9 tumors) or both agents (*n* = 9 tumors) 3 times/week over 2 weeks. (**C**) Tumor growth curves showing the increase in tumor size for each treatment group. Data are presented as mean and SEM. (**D**) Quantification and statistical analysis (Student's *t*-test) of the tumors among different treatment groups 19 days after tumor implantation. (**E**) Representative photographs of the tumors; A = ABT263, W = WP1130. (**F**) Representative microphotographs showing the histological morphology (H & E staining) of tumors from animals receiving treatment either with vehicle or the combination of ABT263 and WP1130. Magnification, ×40; scale bar, 100 μm. (**G**) representative microphotographs showing the histological morphology (H & E staining) of the indicated organs among representative animals receiving treatment either with vehicle or the combination of ABT263 and WP1130. Magnification, ×40; scale bar, 100 μm.

Next, we examined whether inhibition of deubiquitinases exerts anti-cancer activity in a murine orthotopic glioblastoma model. WP1130 or vehicle was administered locally by convection-enhanced delivery. In order to verify correct drug delivery, Gadolinium was added to the solvent in the micro-osmotic pumps and intraparenchymal Gadolinium signal was detected by MRI (Figure 5A). Animals receiving a continuous treatment for 7 days with WP1130 showed a marked prolongation of survival when compared to animals receiving vehicle (Figure 5B). Notably, two WP1130-treated animals were still alive 6 months after tumor cell injection.

### Combined inhibition of deubiquitinases and Bcl-2/Bcl-xL attenuates tumor growth *in vivo*

We showed that a combined treatment with the deubiquitinase inhibitor WP1130 and the BH3-mimetic ABT263 resulted in a synergistic anti-neoplastic activity *in vitro*. Therefore, we next addressed the question whether this therapeutic strategy would also prove to be of benefit *in vivo*. In a heterotopic U251 glioblastoma model, combined treatment with WP1130 and ABT263 resulted in a significant attenuation in tumor growth when compared to single-agent treatment or control (Figure 5C–5E). Moreover, the combination treatment led to a 47.7% regression of tumor size suggesting that this treatment not only attenuated tumor growth but induced tumor cell death. Consistently, histological analysis revealed a significantly reduced cellular density in tumor tissue (Figure 5F). Importantly, any signs for a potential organ toxicity of this therapeutic regimen were absent (Figure 5G).

## DISCUSSION

Deubiquitinases are up-regulated and are pivotal enzymes within cancer cells since they participate in maintaining high levels of molecules that ultimately drive the relentless growth of tumor cells. Certain deubiquitinases have received particular attention as treatment targets. Among these enzymes is the deubiquitinase Usp9X, which has been demonstrated to be overexpressed in various malignant tumors, including breast cancer, lung carcinoma, prostatic carcinoma, colon carcinoma, multiple myeloma, acute lymphoblastic leukemia and follicular and diffuse large B-cell lymphoma [5–9].

In glioblastoma, only little is known about the role of Usp9X and other deubiquitinases. In our report, we confirm earlier observations made in other tumor entities showing that Usp9X levels are up-regulated in tumor tissue but not normal tissue. Thus, it appears that targeting Usp9X might have impact on glioma growth and could be preferentially targeted over normal non-neoplastic tissue. Another recent report supports the relevance of Usp9X in brain tumors [10]. Cox et al. demonstrated that Usp9X is one of the proteins that interacts with the transcription factor SOX2, a marker of human stem cells. They also provided early evidence that interference with Usp9X impairs the growth of established human glioblastoma cells *in vitro* [10]. Our present study extends these findings and provides significant additional insight on the role of Usp9X in glioblastoma biology. We show that the Usp9X inhibitor, WP1130, exerts anti-glioma activity in established, stem cell-like glioma, proneural and patient-derived glioblastoma xenograft cells. Notably, WP1130 is not inhibiting only Usp9X, but also other deubiquitinases such as Usp14 and Usp5 [11]. However, siRNA mediated knock-down of Usp9X in glioblastoma cells recapitulates the effects of WP1130, suggesting that WP1130 significantly mediates its anti-glioma effects by interference with Usp9X function. Our results are in agreement with earlier reports in other tumor types [6, 9, 12–15]. Conflicting results have been reported on the role of Usp9X in pancreatic cancer. Initially, it was claimed that Usp9X interferes with cell death of normal pancreatic cells in a cell death process that was referred to as “zymophagy” [16]. Contrasting these results, it was shown that in conditional knock-out mice the loss of Usp9X paradoxically entertained tumor growth in the presence of mutated Kras (G12D) [17]. Therefore, Usp9X was claimed to exert tumor-suppressive functions [17]. However, in the context of established pancreatic cancer cell line model systems Usp9X appears to have pro-survival functions [12], which is also in line with our findings in various glioblastoma models. Suppression of Usp9X levels reduced the growth of several different pancreatic cancer cell lines, including BxPC3 and others, and consistent with that observation altered the cell cycle profile of these cells [12]. Similarly to the knock-down of Usp9X, the deubiquitinase inhibitor WP1130 exerted anti-proliferative effects on pancreatic cancer cells. Although these observations appear to contradict the results of the earlier studies in transgenic mice, it needs to be acknowledged that the studies in cell lines were conducted *in vitro* and the effects have not been validated in proper *in vivo* model systems.

Mechanistically, the genetic and pharmacological interference with Usp9X leads to a strong activation of apoptosis with initiator- and effector caspase activation in different types of glioblastoma model systems. The involvement of apoptotic cell death is in agreement with other recent studies that have shown that Usp9X loss-of function activates or enhances apoptosis [4, 7, 9, 18–21]. For instance, in the setting of B-cell acute lymphoblastic leukemia (B-ALL) interference with Usp9X caused bax-dependent apoptosis and suppression of the pro-survival mTORC1 signaling pathway [9]. With respect to other forms of cell death, a recent study demonstrated that WP1130 inhibited autophagy through inhibition of ULK1, which may suggest that Usp9X inhibition blocks cytoprotective autophagy in order to enhance apoptosis [22].

Given the engagement of apoptotic cell death upon treatment with WP1130, we hypothesized that the intrinsic apoptotic signaling cascade is regulated by loss of Usp9X function. In line with this notion, increasing concentrations of WP1130 depleted the levels of certain reported Usp9X targets, such as Mcl-1 [7, 8] and the inhibitor of apoptosis protein, Survivin [23]. However, other anti-apoptotic molecules, such as Bcl-2, Bcl-xL and XIAP, were down-regulated as well, while the pro-apoptotic Noxa was increased. However, it is important to bear in mind that due to overlapping effects of caspase activation, one cannot assume that all findings made after WP1130 treatment are a consequence of Usp9X inhibition. So far, the increase of pro-apoptotic Noxa has not been described in the context of Usp9X inhibition and this will likely further enhance susceptibility to intrinsic apoptosis. Overall, these observations reinforce the notion that interference with the Usp9X signaling axis confers a pro-apoptotic state in various glioblastoma cell culture models. These findings are in line with previous reports [10, 18, 19].

Since we observed a significant modulation of Mcl- 1 and Noxa expression, we tested the hypothesis that interference with Usp9X signaling renders cells sensitive to Bcl-2/Bcl-xL inhibition by the BH3-mimetic, ABT263. To that end, we found that pharmacological inhibition of Usp9X sensitized patient-derived xenograft cells (GBM12) to the cytotoxic effects of ABT263 and that this effect is synergistic. Moreover, we found that mechanistically this effect was linked to WP1130-mediated suppression of Mcl-1 protein levels, since Mcl-1 is a major factor of resistance towards BH3-mimetic-mediated apoptosis [24–30]. Along these lines, it is conceivable that Noxa up-regulation caused by Usp9X inhibition might contribute to the sensitization to BH3-mimetic cell death given the pro-apoptotic effects of Noxa on Mcl-1 [31–33]. Our findings are in line with previous observations in pancreatic cancer, in which cell death by another BH3-mimetic was enhanced by blocking the binding of Usp9X to Mcl-1 *in vitro* and *in vivo* [34]. However, even though WP1130-mediated down-regulation of the Usp9X/Mcl-1 axis appears to contribute to the enhancement of the pro-apoptotic effect of ABT263 it is very likely that other molecular alterations following treatment with WP1130 add to this effect as well.

Given our observations that loss-of Usp9X function impaired expression of 1) the anti-apoptotic Bcl-2 family member Mcl-1, which has been previously shown to enhance TRAIL-mediated apoptosis [35], and 2) also of the IAPs, Survivin and XIAP [36, 37] we determined whether antagonism of Usp9X function also primes glioblastoma cells to the cytotoxic effects of the death ligand TRAIL [37–43]. Our findings showed that interference with Usp9X signaling rendered glioblastoma cell cultures sensitive to TRAIL-mediated apoptosis, which to the best of our knowledge has not been reported thus far for any tumor entity. Considering our observations with respect to Bcl-2 family members and IAPs, Usp9X appears to block death-receptor-mediated apoptosis most likely at the level of initiator- as well as effector caspases, involving a combination of factors. It appears less likely that the endogenous inhibitor of caspase 8, c-FLIP [44], which akin to Survivin and Mcl-1 is a molecule with a relatively short half-life and is stabilized by chaperones and potentially other deubiquitinases, is involved in TRAIL/WP1130-mediated cell death, because an interaction of Usp9X and c-FLIP has not been proven [45, 46]. In fact, c-FLIP served as a negative control for its inability to bind to Usp9X [8]. A drawback of targeting TRAIL-mediated apoptosis in glioblastoma is represented by findings that suggest that certain death-receptors and caspase 8 levels are silenced in a fraction of glioblastomas [47–49]. Despite some controversy, targeting death-receptor-mediated apoptosis appears to be still a viable option for the treatment of glioblastoma, since recent results suggest that a small-molecule inducer of TRAIL (ONC201/TIC10), which is capable to cross the blood brain barrier, prolongs animal survival singly and in combination with bevacizumab [50]. Other approaches utilized stem cells that home to brain tumors and secrete TRAIL, leading to suppression of glioma growth *in vivo* [51].

Finally, we determined as to whether Usp9X signaling is important *in vivo*. For that purpose, we utilized a local-delivery pump system [52] to administer WP1130 to intracranial proneural glioblastomas (*PDGF+*, *PTEN*−*/*−, *TP53*−*/*−). We found that animals receiving WP1130 lived longer, suggesting potential feasibility of WP1130 as an anti-glioma drug. To the best of our knowledge, our observation appears to be the first one that highlights the potential applicability of WP1130 as an anti-glioma compound. We further tested whether WP1130 might also be used in drug combination therapies *in vivo* and found that WP1130 enhanced the effects of ABT263 *in vivo* without any detectable toxicity, though more thorough analyses including hematological, renal and hepatic function panels need to be done by further studies. Although WP1130 has been studied in drug combination in other tumor entities, such as the combination of Prednisone and WP1130 in B-ALL, our report is the first one that demonstrates that WP1130 might be used in combination with other drugs in model systems of glioblastoma. A recent study in multiple myeloma modified the chemical structure of WP1130 and presented a reagent that appeared to have stronger anti-myeloma effects than WP1130 [7], suggesting that this modified compound may bear stronger growth inhibitory effects on glioblastoma models compared to WP1130. Whether treatment with WP1130 would also enhance the anti-neoplastic activity of death-promoting ligands such as TRAIL in glioblastoma models *in vivo* seems likely, but remains to be validated by future studies.

Taken together, our findings highlight that targeting Usp9X for glioblastoma is feasible and that inhibition of Usp9X alone or along with other compounds significantly impairs the growth of preclinical models of glioblastoma *in vitro* and *in vivo*.

## MATERIALS AND METHODS

### Ethics statement

All procedures were in accordance with Animal Welfare Regulations and approved by the Institutional Animal Care and Use Committee at the Columbia University Medical Center. The study was reviewed and approved by the institutional review board at the Columbia University Medical Center.

### Reagents

ABT263 was purchased from ChemieTek (Indianapolis, IN, U.S.A.). WP1130 was purchased from Selleckchem (Houston, TX, U.S.A.). A 10 mM working solution in dimethylsulfoxide (DMSO) was prepared for both reagents prior to storage at −20°C.

### Cell cultures and growth conditions

LN229 (*TP53* mut, *PTEN* wt) and T98G (*TP53* mut, *PTEN* mut) [53] human glioblastoma cells were obtained from the American Type Culture Collection (Manassas, VA, U.S.A.). U251 (*TP53* mut, *PTEN* mut) glioblastoma cells were kindly provided by Dr. James Goldman (Columbia University, New York, NY, U.S.A.). NCH644 and NCH421K stem cell-like glioma cells were obtained from Cell Line Services (CLS, Heidelberg, Germany) [48, 54, 55]. The identities of the glioblastoma cell lines we purchased were confirmed by the respective source of purchase. SF188 (*TP53* mut, *PTEN* wt) [53] pediatric glioblastoma cells were kindly provided by Dr. Craig Thompson (Memorial Sloan Kettering Cancer Center, New York, NY, U.S.A.). MGPP-3 (PDGF(+), p53(−/−), PTEN(−/−)) is a murine proneural glioblastoma cell which was kindly provided by Dr. Peter Canoll (Columbia University, New York, NY, U.S.A.). GBM12 (*TP53* mut, *PTEN* wt) human, patient-derived glioblastoma primary cultures were kindly provided by Dr. Jann Sarkaria (Mayo Clinic, Rochester, MI, U.S.A.) *via* Dr. James Angelastro (University of California, Davis, CA, U.S.A.). All cells were cultured as previously described [56]. Briefly, LN229, U251, T98G and MGPP-3 cells were cultured in DMEM with 10% FBS, 4.5 g/L glucose, 4 mM L-glutamine, 1 mM pyruvate, 100 units/ml penicillin and 100 μg/ml streptomycin for maintenance. For experimental conditions these cells were cultured in DMEM containing only 1.5% FBS to mimick the nutrition starved environment within tumors. For the culture of SF188 the fore-mentioned medium was supplemented in addition with 2 mM L-alanyl-L-glutamine (GlutaMAX^™^-I, Gibco, Japan). NCH644 and NCH421K glioma stem-like cells were cultured in MG-43 medium (CLS, Heidelberg, Germany) for both maintenance and experiments. GBM12 were cultured as described before [57].

### Cell viability assays

In order to examine cellular proliferation, 3-[4, 5-dimethylthiazol-2-yl]-2, 5-diphenyltetrazolium bromide (MTT) assays were performed as previously described [58, 59].

### Measurement of apoptosis and mitochondrial membrane potential

For annexin V/propidium iodide (PI) staining the FITC Annexin V Apoptosis Detection Kit I (BD Pharmingen, U.S.A.) was used according to the manufacturer's instructions. Staining for PI was performed as previously described [58]. The data were analysed with the FlowJo software (version 8.7.1; Tree Star, Ashland, OR, U.S.A.).

### Real-time PCR and cDNA synthesis

RT-PCR was performed as described before (Pareja et al.) using the following primers: Usp9X forward: GTG TCA GTT CGT CTT GCT CAG C; Usp9X reverse: GCT GTA ACG ACC CAC ATC CTG A; Mcl-1 forward: CCA AGA AAG CTG CAT CGA ACC AT; Mcl-1 reverse: CAG CAC ATT CCT GAT GCC ACC T; GAPDH forward: GTC TCC TCT GAC TTC AAC AGC G and GAPDH reverse: ACC ACC CTG TTG CTG TAG CCA A.

### Western blot analysis

Specific protein expression in cell lines was determined by Western blot analysis as described before [18] using the following primary antibodies: Mcl-1 (1:500; CST: Cell Signaling Technology, Danvers, MA), human caspase 9 (1:1,000; CST), cleaved caspase 3 (1:250; CST), cleaved PARP (Asp214, 1:1000; CST), Bcl-xL (1:500; CST), Usp9X (1:1000; CST), XIAP (1:1000; CST), Survivin (1:1000; CST), BIM (1:500; CST), Noxa (1:500, clone 114C307; Calbiochem), β-actin (1:2,000, clone AC15; Sigma Aldrich), Bag3 (1:500; Abcam, Cambridge, MA). 14-3-3 (1:1,000, SCB: Santa Cruz Biotechnology), GAPDH (1:1000, clone 1D4, Novus Biologicals) and secondary HRP-linked antibodies were purchased from SCB.

### siRNA transfection

SignalSilence^®^ Usp9X siRNA I #6308 was purchased from CST. Non-targeting siRNA-pool (ON-TARGETplus Non-targeting Pool, # D-001810-10-05) and Mcl-1 (SMARTpool: ON-TARGETplus Mcl-1 siRNA, L-004501-00-0005) were purchased from Thermo Fisher Scientific (Pittsburgh, PA) and transfected as previously described [43, 60]. Briefly, cells were incubated for 6 h with the formed complexes of Lipofectamine^®^ 2000 (Invitrogen, Carlsbad, CA, U.S.A.) and the respective siRNA (12-well condition) in DMEM without FBS and antibiotics. After 6 h, FBS was added to a total concentration of 1.5%.

### Orthotopic xenograft and convection-enhanced delivery model

MGPP-3 murine glioblastoma xenografts were stereotactically implanted into 6- to 8-week-old male or female mice (Nu/Nu GFP) weighing 20 to 30 g as previously described [56, 61]. The study was approved by the Institutional Animal Care and Use Committee at the Columbia University Medical Center. For the generation of intracranial tumors, 25000 cells in 2 μL PBS (Gibco, Life Technologies), were implanted into the right basal ganglia of anesthetized mice using a small animal stereotactic frame (Stoelting). Motorized injections were performed at a rate of 0.3 μL/min.

Convection-enhanced delivery was performed as described before [61, 62]. Five days after implantation of tumor cells, alzet^®^ micro-osmotic pumps (DURECT Corporation, Cupertino, CA, U.S.A.) were implanted for a 7-day long continuous intracranial infusion of WP1130 or solvent. To verify intracranial drug administration 1% Gadolinium was added to the pumps (Omniscan, GE Healthcare Inc., Princeton, NJ, U.S.A.) and MRIs (Bruker ICON^™^, 1 Tesla) were performed after removal of the pump system. Survival was assessed by calculating Kaplan–Meier curves.

### Subcutaneous xenograft model

1 × 10^6^ U251 cells suspended 1:1 in Matrigel^®^ Matrix (Corning Inc., Corning, NY, U.S.A.) were implanted subcutaneously into the flanks of 6-8 week-old SCID SHO mice as previously described [18]. Treatment was performed intraperitoneally 3 times a week for 2 weeks. For intraperitoneal application ABT263 and WP1130 were dissolved in 80% Cremophor EL (SIGMA, St. Louis, MO) and 20% Ethanol (Pharmco-Aaper, Brookfield, CT) (v/v).

### Histological analysis

Subcutaneous tumors and samples from organs were extracted from SCID SHO mice and fixed for at least 24 h in 10% PBS-buffered formalin [19]. Then tissues were embedded in paraffin and 4 μm thick sections were cut prior to staining with hematoxylin and eosin. Microphotographs were taken at ×40 magnification.

### Statistical analysis

Statistical significance was assessed by Student's *t*-test using Prism version 5.04 (GraphPad, La Jolla, CA, U.S.A.). A *p* ≤ 0.05 was considered statistically significant. The CompuSyn software (ComboSyn, Inc., Paramus, NJ, U.S.A. – www.combosyn.com last accessed 06/01/15) was used for the drug combination analysis including the calculation of the combination index (CI) and isobologram. A CI < 1 was considered as synergistic, a CI = 1 as additive and a CI > 1 as antagonistic. The concentration for each compound resulting in 50% inhibition (ED_50_) is normalized to 1, plotted on x- or y-axis and connected by a line which represents the ED_50_ isobologram. Data points of drug combinations plotted below the connecting line represent a synergistic interaction, data points located on the line represent an additive interaction and data points located above the connecting line represent an antagonistic interaction.

## SUPPLEMENTARY MATERIALS FIGURES


