# Supplementary material for: Inhibition of deubiquitinases primes glioblastoma cells to apoptosis in vitro and in vivo
Source: Oncotarget. 2016 Feb 10;7(11):12791–805. doi: 10.18632/oncotarget.7302 (PMC4914322; doi:10.18632/oncotarget.7302)
Supplement: Supplementary file 1 [file oncotarget-07-12791-s001.pdf]

Inhibition of deubiquitinases primes glioblastoma cells to apoptosis *in vitro* and *in vivo*

Supplementary Materials

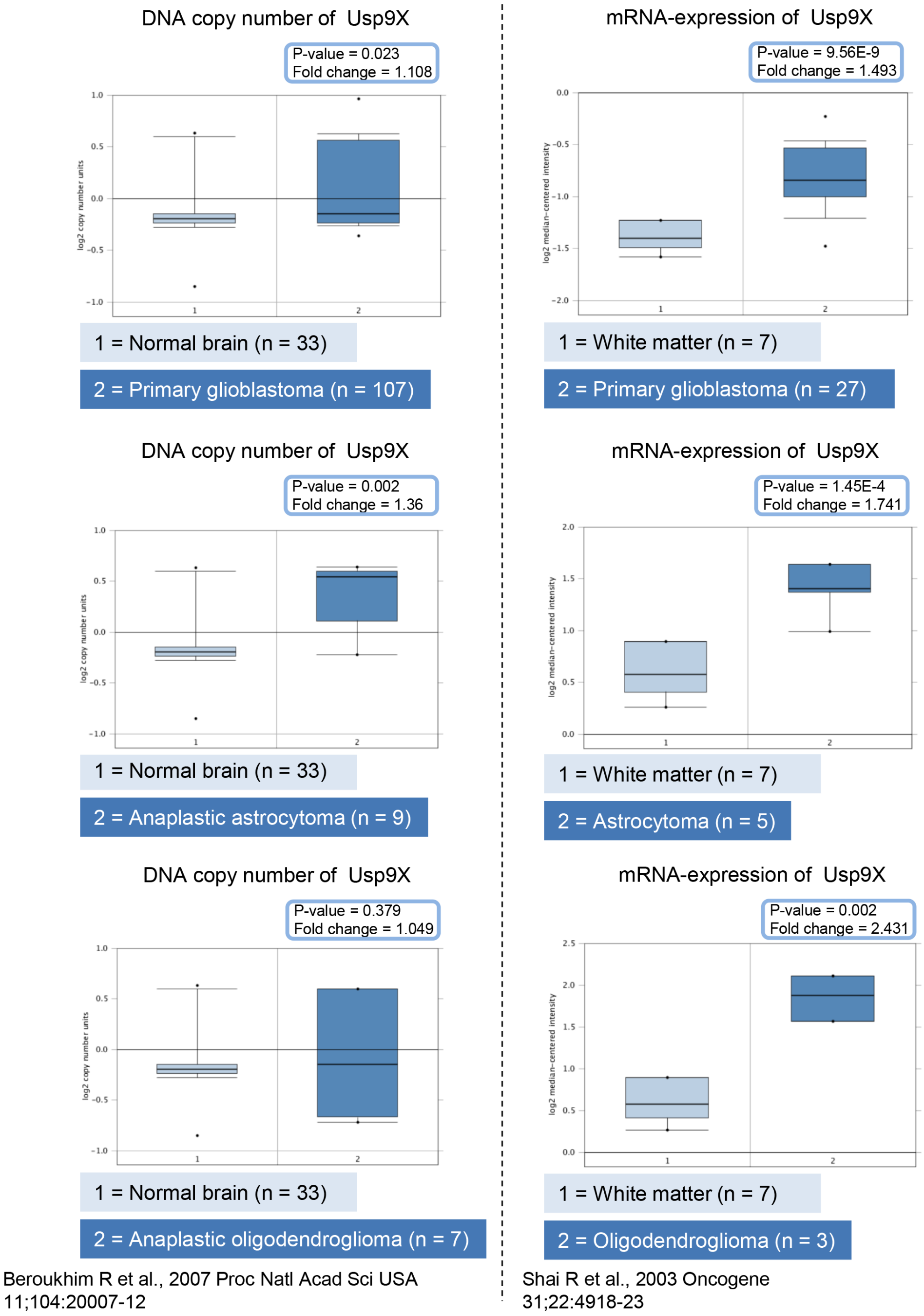

Supplementary Figure S1: Usp9X DNA copy number and mRNA expression in different gliomas compared to normal brain. *In silico* gene expression analysis of Usp9X using the oncomine® database (www.oncomine.org, 11/2015, Compendia Bioscience, Ann Arbor, MI).

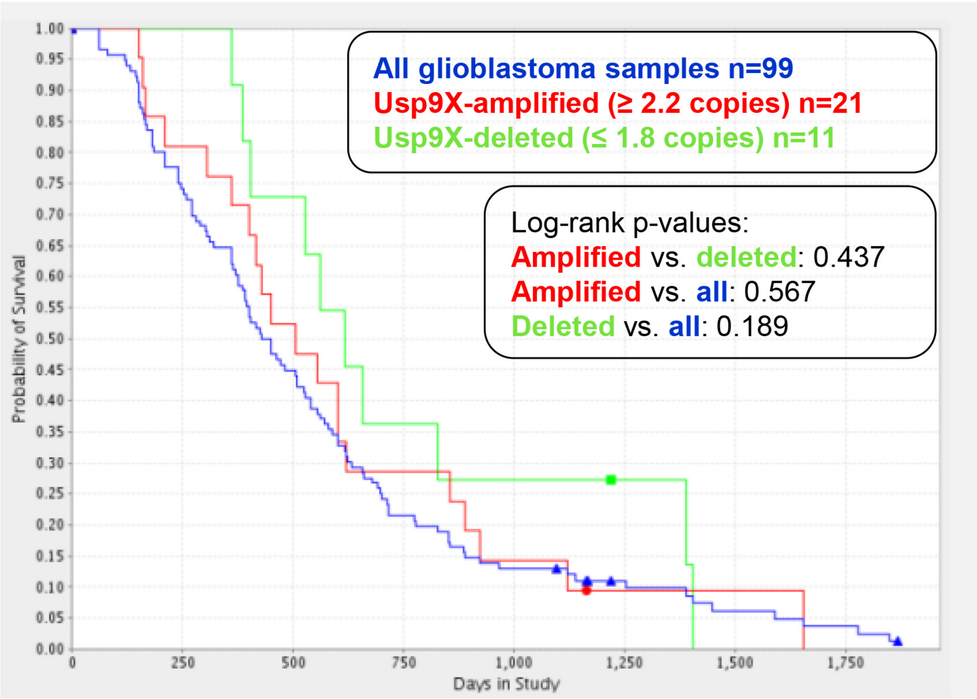

Supplementary Figure S2: *In silico* analysis on the survival of glioblastoma patients based on the amplification status of the Usp9X gene (National Cancer Institute. 2005. REMBRANDT home page. <http://rembrandt.nci.nih.gov>).

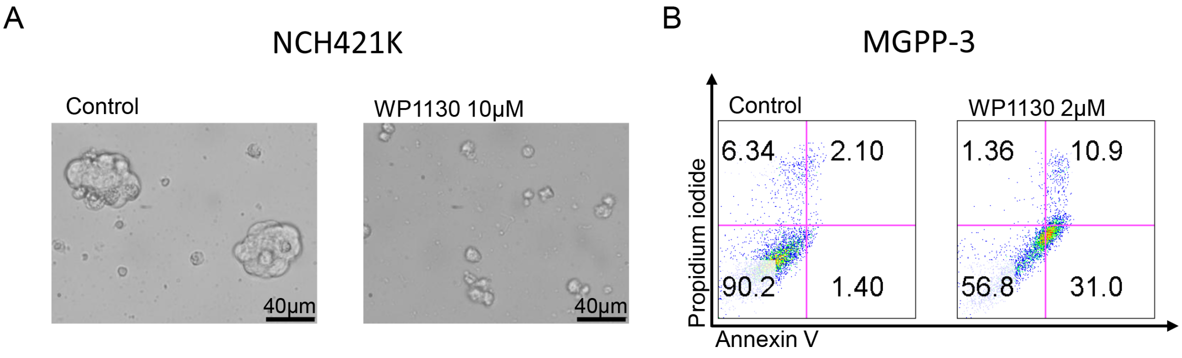

Supplementary Figure S3: (**A**) Representative microphotographs of NCH421K glioma stem-like cells treated with solvent or WP1130 for 48 h at indicated concentrations. Magnification, ×40; scale bar, 40 µm. (**B**) representative flow plots of MGPP-3 glioblastoma cells that were treated as indicated with WP1130 prior to staining with annexin V/propidium iodide and flow cytometric analysis.

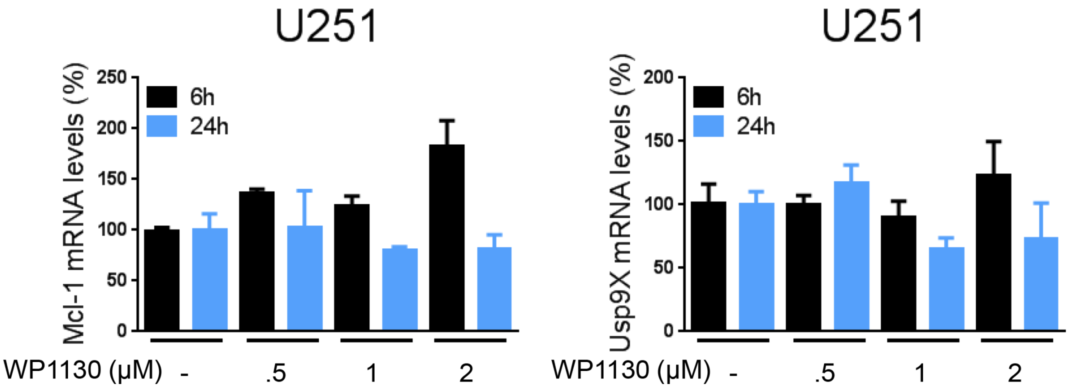

Supplementary Figure S4: U251 glioblastoma cells were treated for 6 h or 24 h with increasing concentrations of WP1130 prior to isolating RNA, reverse transcription and real-time PCR analysis for Mcl-1 and Usp9X. Columns: means. Bars: SD.
